# Community awareness and health providers’ perspectives on zoonotic *Plasmodium knowlesi* malaria in Thailand: A mixed-methods assessment

**DOI:** 10.1371/journal.pntd.0013891

**Published:** 2026-02-18

**Authors:** Piyarat Sripoorote, Nichakan Inthitanon, Yupaporn Wattanagoon, Liwang Cui, Wang Nguitragool, Kimberly Fornace, Jetsumon Sattabongkot, Daniel M. Parker, Pyae Linn Aung

**Affiliations:** 1 Mahidol Vivax Research Unit, Faculty of Tropical Medicine, Mahidol University, Bangkok, Thailand; 2 Department of Clinical Tropical Medicine, Faculty of Tropical Medicine, Mahidol University, Bangkok, Thailand; 3 Division of Infectious Diseases and International Medicine, Department of Internal Medicine, Morsani College of Medicine, University of South Florida, Tallahassee, Florida, United States of America; 4 Department of Molecular Tropical Medicine and Genetics, Faculty of Tropical Medicine, Mahidol University, Bangkok, Thailand; 5 Saw Swee Hock School of Public Health and National University Health System, National University of Singapore, Singapore, Singapore; 6 Department of Population Health and Disease Prevention, Department of Epidemiology & Biostatistics, University of California, Irvine, California, United States of America; University of the Witwatersrand Johannesburg, SOUTH AFRICA

## Abstract

**Background:**

*Plasmodium knowlesi* poses an emerging challenge for malaria control in Southeast Asia due to its zoonotic nature, diagnostic complexity and lack of species-specific control strategies. These factors complicate both prevention and case management efforts. Understanding both community awareness and healthcare provider perspectives is critical for informing targeted responses. This study aimed to assess awareness of *P. knowlesi* malaria among at-risk community members in southern Thailand and to explore contextual challenges through qualitative insights from healthcare providers, including provincial officers, district-based malaria control staff, and village health volunteers.

**Methods:**

An explanatory sequential mixed-methods design was employed between April and May 2025. A cross-sectional survey of 300 adults residing in three *P. knowlesi*-endemic districts was conducted using a structured questionnaire that included eight items assessing overall awareness. Descriptive statistics, violin plots, and multivariable generalized linear modeling were used to identify factors associated with awareness scores. Subsequently, semi-structured interviews were conducted with 28 healthcare providers across various administrative levels. Qualitative data were thematically analyzed.

**Results:**

Overall awareness of *P. knowlesi* malaria was moderate (mean score: 10.7/23; SD ± 2.9; range: 3.0–20.0). Female individuals, living farther from health facilities, receiving malaria-related health education, and having higher attitude scores were significantly associated with increased awareness (*p* < 0.05). Healthcare providers reported a decline in *P. knowlesi* incidence, alongside persistent transmission risks in forested areas. Health education efforts were largely reactive, delivered following case detection, but often included information about monkey-to-human transmission.

**Conclusions:**

Awareness of *P. knowlesi* malaria remains suboptimal among high-risk populations, particularly among men, those who had received limited health education, and individuals with poor attitudes toward malaria. Integrated, proactive, and male-focused health promotion strategies are essential to enhance community knowledge and support disease control efforts.

## Author summary

Zoonotic malaria caused by *Plasmodium knowlesi*, a parasite naturally carried by monkeys, is an emerging public health concern in Southeast Asia. In Thailand, the number of reported *P. knowlesi* cases has increased in recent years, particularly in forested areas near the borders with Malaysia and Myanmar. However, little is known about how well at-risk communities understand this disease or how prepared the healthcare system is to respond. This study is the first in Thailand to explore both community awareness and healthcare provider perspectives on *P. knowlesi* malaria. Using a mixed-methods approach, we surveyed 300 adults living in areas with known *P. knowlesi* cases and interviewed 28 healthcare workers involved in malaria control. We found that community awareness was moderate, with women and those who had received malaria-related health education showing higher scores. Healthcare providers reported operational challenges, such as delayed diagnosis and limitations of current control strategies that were not designed for zoonotic transmission. Our findings highlight the need for targeted health education, especially for men and forest-exposed populations, and improved strategies to address the unique risks of monkey-to-human malaria transmission. These results can help inform national policies as Thailand moves toward its malaria elimination goals.

## 1. Introduction

In 2023, the World Health Organization (WHO) Southeast Asia (SEA) Region accounted for approximately 1.5% of global malaria cases, with eight countries still experiencing ongoing transmission [1]. While the Maldives and Sri Lanka have achieved malaria-free status, several other countries are making progress toward the regional elimination target of 2030. However, recent increases in the number of malaria cases have been noted in India, Indonesia, and Myanmar [1]. Similarly, in Thailand, despite sustained progress over the past decade, the country experienced a resurgence in malaria, with the national annual parasite index (API) increasing from approximately 0.13 per 1,000 population in 2021 to 0.65 per 1,000 in 2023, representing an approximate five-fold rise [1,2]. Transmission remains highly heterogeneous. Many central and eastern provinces report APIs near zero, while provinces bordering Myanmar and Malaysia contribute the majority of cases [2]. These regions are home to high-risk populations, such as forest-goers and forest-adjacent communities, who frequently engage in outdoor activities without taking protective measures like using insecticide-treated nets (ITNs), thereby contributing to sustained transmission [3,4].

An emerging challenge to malaria control and elimination in SEA is *Plasmodium knowlesi*, a zoonotic malaria parasite transmitted to humans through mosquito vectors, with species of macaques serving as its natural hosts [5,6]. In Thailand, reported *P. knowlesi* cases rose from 13 in 2020–275 in 2023, corresponding to an increase from approximately 0.004 to 0.39 cases per 1,000 population in affected districts. Although *P. knowlesi* still represents roughly 3% to 5% of all confirmed malaria cases nationally, infections remain predominantly concentrated in border provinces [2]. Other non-human primate plasmodial species, including *Plasmodium cynomolgi*, have also been reported in Thailand, further complicating elimination efforts [6]. First identified as a human pathogen in Malaysia in 2004, *P. knowlesi* has since become recognized as the only indigenous cause of human malaria, with reported cases having increased more than 100-fold by 2021 [1,7]. The parasite presents unique diagnostic and treatment challenges: its morphology closely resembles *P. malariae* under microscopy, but unlike *P. malariae* infection it may lead to rapid parasitemia and severe disease outcomes if not promptly detected and treated [8,9]. Although *P. knowlesi* remains sensitive to artemisinin-based therapies and chloroquine, clinical management is complicated by the parasite’s rapid 24-hour erythrocytic cycle, which increases the risk of sudden clinical deterioration [9]. Thus, delayed or missed diagnosis, rather than drug resistance, represents the main treatment challenge. In response to these risks, the 2022 WHO Global Malaria Elimination Guidelines require countries to demonstrate negligible risk from all species of *Plasmodium*, including zoonotic types such as *P. knowlesi*, to qualify for elimination certification [10].

Transmission is linked to the long-tailed macaque (*Macaca fascicularis*) and pig-tailed macaque (*M. nemestrina*), with vectors belonging to the *Leucosphyrus* group of *Anopheles* mosquitoes facilitating zoonotic spillover [6,11]. Although sustained human-to-human transmission has not been confirmed, people working or residing in forested areas are at heightened risk from exposure to mosquito vectors capable of transmitting the parasite from macaques [12]. Current malaria control strategies, such as use of long-lasting insecticidal nets (LLINs), are effective in household settings, but are often impractical in forest environments. Personal protection methods, such as using mosquito repellents, are important, but adherence to regular use is limited, and their efficacy is generally short-lived, requiring regular reapplication [13]. Critically, no available strategies can reduce transmission from macaques to mosquitoes, and standard vector control tools are insufficient to address the zoonotic reservoir [14]. This highlights the need for species-specific strategies and forest-targeted interventions to support elimination goals. In Thailand, the national malaria control program has implemented a range of social and behavior change interventions, including community-based health education delivered by village health volunteers, targeted outreach in high-risk border zones, and periodic mass media campaigns promoting insecticide-treated net use and early treatment-seeking [15–17]. While these approaches have played an important role in reducing *Plasmodium falciparum* and *Plasmodium vivax* transmission, their adaptation to zoonotic malaria parasites such as *P. knowlesi* has been limited, and existing educational tools rarely address monkey-to-human transmission dynamics.

Evidence from Malaysia suggests that community awareness of *P. knowlesi* malaria remains insufficient. One study reported that 81.7% of individuals in high-risk areas had inadequate knowledge of prevention strategies [18]. Furthermore, risk perception and behavioral responses to monkey encounters, as well as sociodemographic characteristics such as age, have been linked to malaria awareness levels [19,20]. In Thailand, available studies on general malaria awareness, although not specific to *P. knowlesi*, indicate moderate understanding of prevention and treatment, with notable gaps among forest-goers, migrant populations, and residents of remote border areas who face the greatest exposure risks [21–24]. Despite these findings, social and behavioral assessments specific to *P. knowlesi* remain limited, particularly outside Malaysia. As of June 2025, no peer-reviewed studies have assessed *P. knowlesi*-specific awareness in the Thai context. Furthermore, there is ongoing uncertainty across the region about whether the observed rise in *P. knowlesi* cases reflects genuine emergence driven by environmental or ecological change or is largely attributable to improved awareness and the increased use of species-specific diagnostics. This is a key question for Thailand, where case numbers remain relatively low but are increasing. Clarifying this distinction will inform whether further increases in the *P. knowlesi* burden should be anticipated. To address these knowledge gaps, we conducted a study in southern Thailand. The objectives were to (1) quantify awareness toward *P. knowlesi* malaria among at-risk community members and (2) explore operational challenges and contextual barriers through qualitative insights from healthcare providers.

## 2. Methods

### 2.1. Ethics statement

The study protocol was reviewed and approved by the Ethics Committee for Human Research, Faculty of Tropical Medicine, Mahidol University, Bangkok, Thailand (MUTM 2025-012-01). Informed consent forms were signed by all respondents prior to the data collection.

### 2.2. Study design

This study employed a cross-sectional, mixed-methods approach using an explanatory sequential design from April to May 2025, in which qualitative findings were used to contextualize and expand upon results from the preceding quantitative component.

### 2.3. Sampling

#### 2.3.1. Setting.

**Fig 1:**
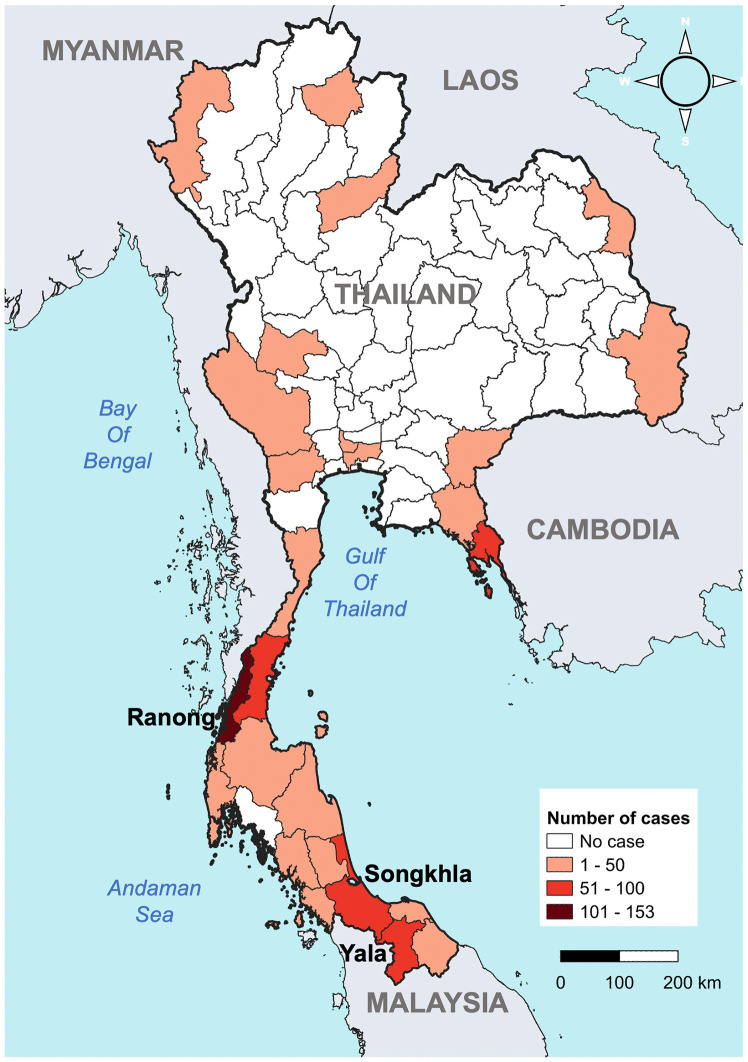
Geographic location of the three study sites in southern Thailand, overlaid on the distribution of *P. knowlesi* cases in Thailand, 2016–2024. Administrative boundary shapefiles were obtained from GADM (Global Administrative Areas; https://gadm.org), accessed through the DIVA-GIS database (https://diva-gis.org), and modified using QGIS software (version 3.34.2-Prizren).

By December 2024, *P. knowlesi* malaria cases had been reported in 27 of 76 provinces across Thailand, with the highest numbers occurring in provinces adjacent to Malaysia and additional clusters identified along the Thailand–Myanmar border [2]. Three southern provinces with the highest reported incidence of *P. knowlesi* malaria were purposively selected: Ranong, Songkhla, and Yala (**Fig 1**). In 2023, Ranong reported 51 cases, Songkhla 33 cases, and Yala 42 cases [2]. These sites were chosen to reflect geographic and epidemiologic diversity: Ranong shares a border with Myanmar, while Songkhla and Yala are adjacent to Malaysia. Macaque populations have also been observed in forested areas near all three provinces, suggesting potential ecological overlap with human settlements [25]. Within each province, two high-incidence villages were selected (six in total), based on 2024 surveillance data and in consultation with provincial public health authorities. Villages near forested zones were prioritized due to their higher potential exposure risk to zoonotic malaria transmission.

#### 2.3.2. Quantitative component.

The target population for the quantitative component consisted of adult community members residing in risk areas for *P. knowlesi* malaria.

Sample size calculations were based on a presumed awareness level of 18.3%, derived from a previous study conducted in Peninsular Malaysia [18]. Using a single population proportion formula for an infinite population [26] and accounting for a 20% non-response margin, a minimum of 276 participants was determined to be sufficient to address the study objectives.

Approximately 100 participants were recruited from each study province, equating to around 50 individuals per village (n = 300). Eligible participants were adults aged 18 years or older who had lived in the village for at least one year. Preferentially, the household head or a knowledgeable representative was chosen. This approach helped ensure that respondents could reliably report household-level information; however, it may also have influenced awareness and attitude scores, as household heads or knowledgeable adults may be more engaged in community activities and may have greater exposure to health information than other household members. Individuals visibly under the influence of alcohol or drugs at the time of the interview were excluded.

Households were selected using a systematic sampling approach from a village listing provided by local healthcare staff. The starting point was randomly determined via lottery, and one eligible individual was invited to participate in a structured face-to-face interview.

#### 2.3.3. Qualitative component.

The qualitative component was designed to explore stakeholders’ perspectives on the preparedness of the healthcare system in response to the rising incidence of *P. knowlesi* malaria. Healthcare personnel were intentionally recruited from different tiers of the health system, including provincial officers, district-based malaria control staff, and village health volunteers (VHVs).

Yala and Songkhla provinces fall under the same Office of Disease Prevention and Control (ODPC), while Ranong is served by a separate ODPC. From each ODPC, an average of two personnel were recruited. Additionally, each province contains a Vector-Borne Disease Center (VBDC), from which at least two staff were included. Malaria clinics or Vector-Borne Disease Units (VBDUs) located in high-burden districts, Bannangsata (Yala), Rattaphum (Songkhla), and Mueang Ranong (Ranong), were also selected, with two healthcare providers recruited from each facility. At the community level, two VHVs from each of the six villages that also participated in the quantitative study were interviewed. In total, 28 healthcare providers participated in the qualitative inquiry (**S1 Fig**). Participants were eligible if they were 18 years or older, had a minimum of six months of experience working in malaria control within the study areas, and held primary responsibilities related to malaria control. Those whose main duties focused on unrelated health topics were excluded.

### 2.4. Key variables

#### 2.4.1. Quantitative component.

In this study, awareness of *P. knowlesi* malaria was defined as the level of knowledge and understanding community members had about the disease, including its transmission, symptoms, risk factors, prevention, and treatment options. A structured questionnaire in Thai was used to assess this awareness. The original instrument was developed in English, drawing upon previous studies [19,27–29] and expert consultation (**S1 File**). It was then translated into Thai and back-translated by two bilingual malaria experts to ensure linguistic accuracy and cultural appropriateness. As members of the study population were able to speak and understand both Thai and local languages, such as Malay, the Thai version of the questionnaire was used as the primary survey tool.

The questionnaire comprised three key sections. The first section collected socioeconomic and demographic data, including age, sex, educational level, occupation, annual income, household composition, relationship to the household head, length of residence in the village, previous malaria experience, access to malaria control services, and history of receiving malaria-related health education. The second section assessed awareness of *P. knowlesi* malaria through eight targeted items covering disease recognition, causes, preventive measures, treatment-seeking behaviors, and zoonotic transmission. To support the identification of macaques, illustrative images sourced from public online domains were included. The third section explored attitudes toward *P. knowlesi* malaria using a 4-point Likert scale [30], incorporating both positively and negatively worded statements about disease severity, treatment effectiveness, and transmission risks.

Scoring was performed as follows. For the attitude items, positive statements were scored from 4 (strongly agree) to 1 (strongly disagree), while negative statements were reverse-coded. Total attitude scores were derived by adding individual item scores. For the awareness section, each correct answer received a score of 1, and incorrect answers scored 0. The total awareness score represented the sum of all correct responses.

#### 2.4.2. Qualitative component.

The qualitative component aimed to examine stakeholders’ perceptions of current preparedness and response to *P. knowlesi* malaria in light of findings from the quantitative survey. For instance, where community awareness appeared low, participants were asked whether targeted interventions were being implemented and if existing measures were sufficient.

A semi-structured interview guide was developed based on a review of relevant literature, recent studies, and contextual factors specific to the Thai–Malay and Thai–Myanmar border areas [29,31,32]. The guide included four sections. The first collected demographic information such as age, gender, and years of service. The second explored perceptions of *P. knowlesi* trends in the local context. The third focused on the scope and content of current malaria control activities, with particular attention to whether they specifically addressed *P. knowlesi*. The fourth section invited participants to describe implementation challenges, including operational, communication, or resource-related barriers to effective response (**S2 File**).

### 2.5. Procedures

#### 2.5.1. Quantitative component.

In each study district, four to five trained local data collectors, fluent in Thai and familiar with the local dialects, including Malay, were recruited. These individuals received one day of intensive training on study objectives, survey administration, informed consent, and research ethics. Structured interviews were conducted in Thai. Where necessary, interviewers provided informal translation into the local languages, such as Malay, to ensure participant comprehension. Each interview lasted approximately 15 minutes. Field supervision was conducted by members of the core research team to ensure adherence to protocols and to address any emerging issues during data collection.

#### 2.5.2. Qualitative component..

Face-to-face structured interviews with healthcare providers were conducted at health facilities or village health posts. Interviews were held in private locations to maintain confidentiality and minimize interruptions. All interviews were conducted by a PhD-level female researcher with formal training in qualitative methods, social and behavioral research, and malaria fieldwork. During each session, the lead interviewer facilitated the interview while two additional female Thai researchers, also trained in qualitative data collection, took handwritten notes. Each interview lasted approximately 30 minutes.

### 2.6. Statistical analysis

All quantitative analyses were conducted using RStudio version 2025.05.0 + 496 (R Foundation for Statistical Computing, Vienna, Austria). Descriptive statistics were calculated to summarize participant characteristics. Continuous variables were presented as means and standard deviations (SD), along with the minimum and maximum values. Categorical variables were described using frequencies and percentages. To visually illustrate the distribution of awareness and attitude scores, violin plots with overlaid boxplots were generated.

To identify independent factors associated with awareness of *P. knowlesi* malaria, a generalized linear model (GLM) with a Gaussian distribution and identity link function was applied. The full R model specification, including the formula and model code, is provided in **S1 Text**. All potential covariates were included in the model, and village was adjusted for as a fixed effect to control for site-level differences. Village was incorporated as a fixed effect rather than a random effect because only six villages were included, and all were purposively selected based on epidemiologic criteria rather than randomly sampled. Under these conditions, fixed-effect specification provides more stable estimates and avoids unreliable variance component estimation that can occur with very small numbers of clusters. Regression coefficients (β), 95% confidence intervals (CI), and *p*-values were reported. Robust standard errors were calculated using heteroskedasticity-consistent covariance estimators (HC1) to account for potential variance heterogeneity.

Three theoretically justified interaction terms were tested between malaria health education and (1) distance to health facility, (2) education level, and (3) sex by occupation. None were statistically significant and were excluded from the final model. Model diagnostics were conducted to assess the assumptions of linearity, normality of residuals, multicollinearity, and influential observations. Residuals appeared approximately normally distributed and randomly scattered, indicating no major violations of linearity or homoscedasticity. No highly influential observations were identified based on Cook’s distance, and multicollinearity was not detected (all variance inflation factors < 2). The final model was judged to be appropriate for interpretation. Statistical significance was defined as a two-sided *p*-value less than 0.05.

The qualitative component followed the quantitative phase of an explanatory sequential mixed-methods design. Deductive thematic analysis was conducted using NVivo (QSR International, Version 14), guided by three predefined themes (*P. knowlesi* trend, specific control activities, and challenges) that structured the qualitative inquiry. Within each major theme, sub-themes were identified inductively through close reading and iterative coding. To ensure linguistic accuracy and conceptual equivalence, all responses were translated into English by a bilingual researcher with social science expertise and then independently back-translated into Thai by a second researcher unfamiliar with the original transcripts. Discrepancies were discussed and resolved collaboratively. To ensure transparent and balanced reporting, the selection of quotations followed a structured process. After all transcripts were coded, two researchers reviewed all excerpts within each sub-theme and identified quotations based on (i) relevance to the theme, (ii) clarity and expressiveness, (iii) ability to illustrate commonly shared or contrasting viewpoints, and (iv) representation across provider cadres (e.g., public health officers, microscopists, village health volunteers). Preference was given to quotes reflecting patterns observed across multiple interviews rather than isolated comments. Final selections were jointly reviewed to ensure accuracy, balance, and alignment with the overall thematic interpretation. Final themes were interpreted in relation to the study objectives and compared with quantitative findings to identify areas of convergence and divergence, thereby enhancing the validity and contextual understanding of community awareness and operational challenges related to *P. knowlesi* malaria.

## 3. Results

### 3.1. Quantitative component

#### 3.1.1. Sociodemographic characteristics of study participants.

Among the 300 community participants, the mean age was 47 years (SD ± 14), and two-thirds (68.7%) were female. Most had completed primary or secondary education (85.3%), and over half (57.7%) were forest workers. The mean annual household income was approximately 102,000 THB, although this varied widely across individuals. Participants reported living with a mean household size of four members, and just over half (54.0%) were not the head of household. Participants had resided in their communities for a mean period of 28 years, with a mean distance from the nearest health facility of nearly six miles, and a mean travel time of 13 minutes to get to it. A majority (64.0%) reported having had malaria at some point, and nearly 86% had received malaria-related health education. As regards the reported health education, community health workers (67.3%) and village health volunteers (69.6%) were the most common sources (**Table 1**).

**Table 1:** Sociodemographic characteristics of study participants (n = 300).

Characteristic	n (%) or Mean ± SD (Min–Max)
**Age (years)**	47 ± 14 (18–75)
**Sex**	
Female	206 (68.7)
Male	94 (31.3)
**Education**	
No formal education	19 (6.3)
Primary school	124 (41.3)
Secondary school	132 (44.0)
College and above	25 (8.3)
**Occupation**	
Forest workers	173 (57.7)
Non-forest workers^a^	127 (42.3)
**Annual income (THB)**	101858 ± 110481 (3000–900000)
**Household members**	4.09 ± 1.97 (1–12)
**Relationship to household head**	
Household head	138 (46.0)
Non-household head	162 (54.0)
**Length of residence (years)**	28 ± 16 (1–75)
**Distance to health facility (miles)**	5.8 ± 4.8 (0·1–30.0)
**Time to reach health facility (minutes)**	13 ± 8 (1–40)
**Malaria experience**	
Yes	192 (64.0)
No	108 (36.0)
**Received malaria-related health education**	
Yes	257 (85.7)
No	43 (14.3)
**Sources of malaria-related health education (n = 257)** ^**b**^	
Health professionals	55 (21.4)
Community health workers	173 (67.3)
Village health volunteers	179 (69.6)
Radio/TV	39 (15.2)
Internet/social media	53 (20.6)
Family/friends	23 (8.9)

^a^ Includes dependents, housewives, merchants, government employees, etc.; ^b^ Multiple responses allowed; THB = Thai Baht (33 THB ~ 1 USD); SD = standard deviation.

#### 3.1.2. Attitudes toward *P. knowlesi* malaria.

**Fig 2:**
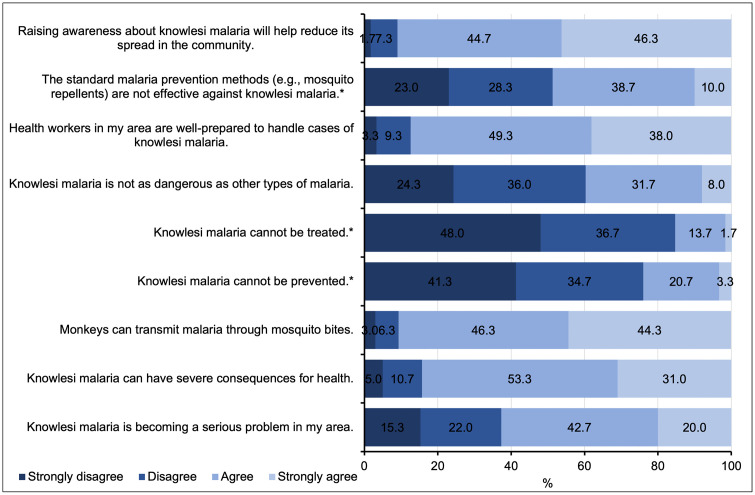
Attitudes toward *P. knowlesi* malaria among community participants (n =300). (* indicates negative statements).

Overall, participants expressed positive attitudes toward the prevention of *P. knowlesi* malaria and understanding of its seriousness (**Fig 2**). Most (91.0%) agreed that raising awareness would help reduce its spread, and 84.3% correctly recognized that monkeys can play a role in the transmission of the disease through mosquito bites. While 84.3% disagreed with the statement that *P. knowlesi* “cannot be treated,” and 76.0% disagreed that it “cannot be prevented,” a substantial proportion still questioned the effectiveness of standard preventive methods (48.7% agreed or strongly agreed). Notably, 42.7% of participants perceived *P. knowlesi* as a growing problem in their area, and 53.3% recognized its potential severity.

#### 3.1.3. Awareness of *P. knowlesi* malaria.

**Table 2** summarizes item-level awareness among participants. Approximately 61.0% had heard of *P. knowlesi* or “monkey malaria,” and over half (54.3%) were aware of recent cases in their communities. Seventy percent had seen macaques near their homes or workplaces. When asked about high-risk groups, the most commonly cited were people living near forests (77.7%), forest workers (59.7%), and farmers (52.0%). Most participants correctly identified mosquito bites (85.7%) as a route of transmission, while a smaller proportion also mentioned forest exposure (26.0%) or contact with monkeys (26.0%). Commonly reported preventive measures included using mosquito nets (86.0%) and repellents (67.7%). However, only 4.3% of participants rated themselves as “very confident” in their understanding of *P. knowlesi* malaria.

**Table 2:** Awareness of *P. knowlesi* malaria among community participants (n = 300).

Awareness item	n (%)
**Have you ever heard of *P. knowlesi* malaria, *Pk* malaria, Monkey malaria, or malaria transmitted from monkeys?**	
Yes	183 (61.0)
No	117 (39.0)
**Are you aware of any recent cases of *P. knowlesi* malaria in your community or nearby regions?**	
Yes	163 (54.3)
No	137 (45.7)
**Have you ever seen long-tailed and pig-tailed macaques around your household or workplace?**	
Yes	210 (70.0)
No	90 (30.0)
**Do you know of any specific groups or activities that are at higher risk of *P. knowlesi* malaria in your area?** ^**a**^	
Forest workers	179 (59.7)
Farmers	156 (52.0)
People living near forests	233 (77.7)
Children	21 (7.0)
Pregnant women	9 (3.0)
Older people	17 (5.7)
I don’t know	4 (1.3)
**Do you consider yourself to be at risk of contracting *P. knowlesi* malaria?**	
Yes	171 (57.0)
No	129 (43.0)
**Do you know how *P. knowlesi* malaria is transmitted?** ^**a**^	
Via mosquito bites	257 (85.7)
Direct contact with monkeys	78 (26.0)
Through contaminated water or food	46 (15.3)
Living/working in the forest	78 (26.0)
Eating bananas/papayas	11 (3.7)
Staying close to malaria patients	60 (20.0)
I don’t know	16 (5.3)
**Do you know any ways to protect yourself from *P. knowlesi* malaria?** ^**a**^	
Using mosquito nets	258 (86.0)
Wearing long-sleeved clothing	127 (42.3)
Applying mosquito repellents	203 (67.7)
Burning rubbish	32 (10.7)
Avoiding forested areas	101 (33.7)
**On a scale of 1–5, how confident are you in your understanding of *P. knowlesi* malaria?**	
1 (Not confident)	36 (12.0)
2	74 (24.7)
3	139 (46.3)
4	38 (12.7)
5 (Very confident)	13 (4.3)

^a^ Multiple responses allowed.

#### 3.1.4. Attitude and awareness scores toward *P. knowlesi* malaria.

**Fig 3:**
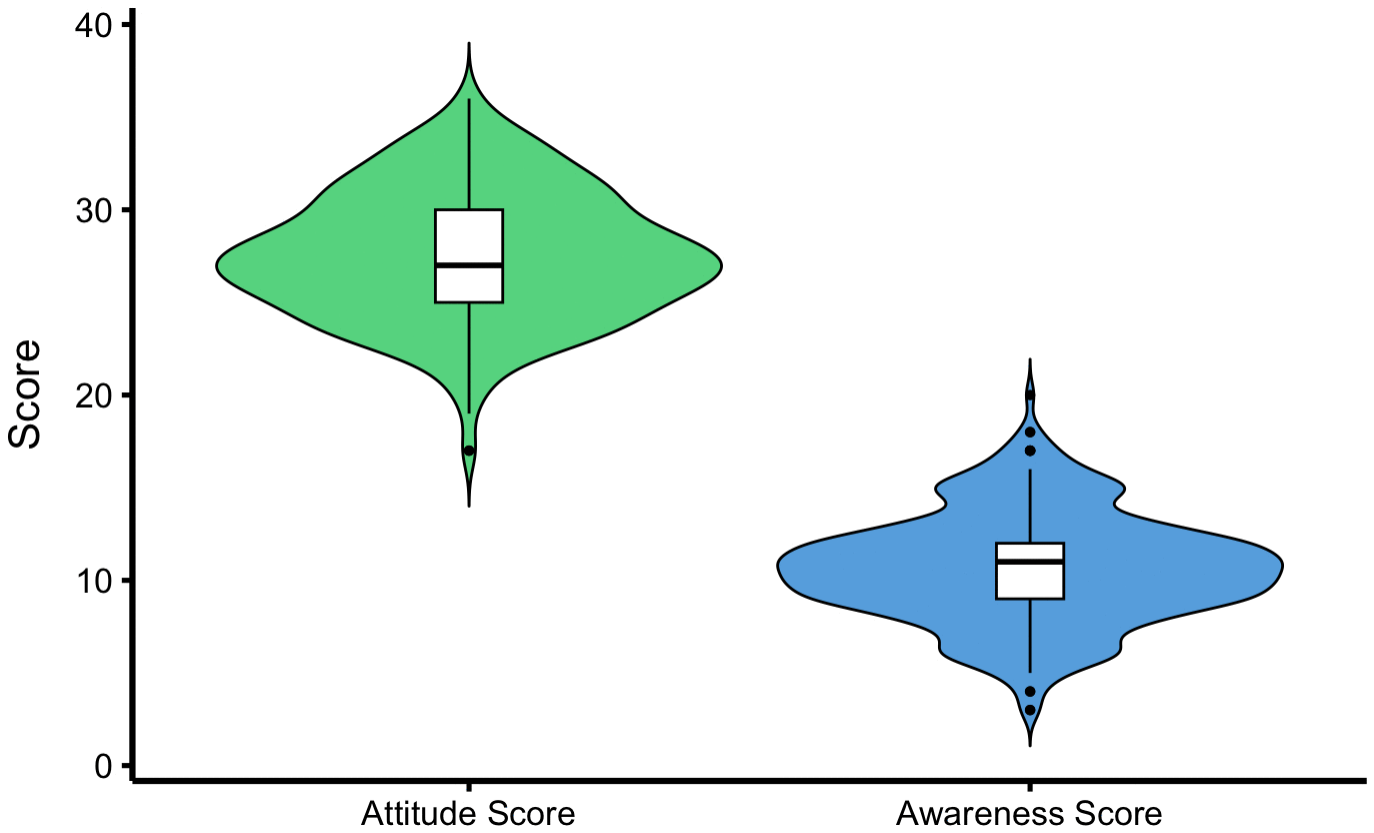
Distribution of attitude and awareness scores toward *P. knowlesi* malaria among community participants. (The attitude (maximum possible score = 36) and awareness (maximum possible score = 23) scales differ in range; therefore, the two distributions are not directly comparable in absolute magnitude, even though presented on a shared axis for simplicity.).

Violin plots with overlaid boxplots illustrated the distribution of total attitude and awareness scores. The mean attitude score was 27.5 (SD ± 3.5; range: 17.0–36.0) out of a maximum possible score of 36. Higher attitude scores indicate more favorable perceptions regarding *P. knowlesi* severity, treatment effectiveness, and the importance of prevention. The mean awareness score was 10·7 (SD ± 2.9; range: 3.0–20.0), based on a maximum score of 23, with higher scores indicating greater knowledge about *P. knowlesi* malaria. Attitude scores showed an approximately symmetrical distribution, while awareness scores were more concentrated toward the lower–middle range, indicating moderate overall knowledge levels among participants (**Fig 3**).

#### 3.1.5. Factors associated with awareness of *P. knowlesi* malaria.

In the multivariable GLM analysis (**Table 3**), male participants had significantly lower awareness scores compared to females (β = –1.1; 95% CI: –1.90 to –0.36; *p* = 0.004). Greater distance to the nearest health facility (β = 0.08; 95% CI: 0.00 to 0.16; *p* = 0.046), receipt of malaria-related health education (β = 1.4; 95% CI: 0.46 to 2.40; *p* = 0.004), and higher attitude scores (β = 0.21; 95% CI: 0.11 to 0.30; *p* < 0.001) were significantly associated with greater awareness. No other variables, including age, income, education, or occupation, showed statistically significant associations.

**Table 3:** Factors associated with overall awareness scores among community participants toward *P. knowlesi* malaria.

Characteristic	β	95% CI	*p*-value
**Age (years)**	0.03	0.00, 0.06	0.092
**Sex**			
Female	Reference		
Male	–1.1	–1.90, –0.36	0.004*
**Education**			
No formal education	–1.5	–3.40, 0.38	0.12
Primary school	–1.4	–2.70, 0.02	0.058
Secondary school	–0.76	–2.00, 0.47	0.200
College and above	Reference		
**Occupation**			
Forest workers	Reference		
Non-forest workers^a^	–0.17	–0.96, 0.63	0.700
**Annual income (per 10,000 THB)**	0.00	–0.04, 0.03	0.800
**Household members**	0.08	–0.10, 0.26	0.400
**Relationship to household head**			
Household head	Reference		
Non-household head	0.07	–0.63, 0.77	0.800
**Length of residence (years)**	–0.01	–0.04, 0.01	0.200
**Distance to health facility (miles)**	0.08	0.00, 0.16	0.046*
**Time to reach health facility (minutes)**	–0.05	–0.09, 0.00	0.055
**Malaria experience**			
Yes	0.53	–0.23, 1.30	0.200
No	Reference		
**Received malaria-related health education**			
Yes	1.4	0.46, 2.40	0.004*
No	Reference		
**Attitude scores**	0.21	0.11, 0.30	< 0.001*

^a^ Includes dependents, housewives, merchants, government employees, etc.; THB = Thai Baht (33 THB ~ 1USD); CI = confidence interval; *Significance at *p*-value < 0.05 by GLM; β = regression coefficient. The model was adjusted for village-fixed effects (with one village as the reference), but none of the village coefficients were statistically significant.

### 3.2. Qualitative component

#### 3.2.1. Participant characteristics.

A total of 28 healthcare providers participated in the qualitative component of this study. Participants included both men (n = 7) and women (n = 21), with ages ranging from 30 to 56 years (median: 47 years). Their length of service in current roles varied widely, from less than two years to over three decades (median: 12 years). Respondents included public health officers, microscopists, malaria post staff, and village health volunteers, offering insights from forested, coastal, and border areas across southern Thailand.

#### 3.2.2. Perceived trends and distribution of *P. knowlesi* malaria.

Healthcare providers consistently reported a declining trend in *P. knowlesi* malaria cases over the past three years. Provinces such as Ranong and Yala noted substantial reductions, with many districts reporting only isolated cases or none at all in recent seasons. This downward trend was attributed to seasonality, heightened community awareness, and strengthened surveillance systems. Nevertheless, providers emphasized that *P. knowlesi* transmission remains associated with forested areas and the presence of macaques, particularly in districts near dense jungle or wildlife reserves. Individuals working in agriculture, forestry, or tourism continued to be considered high-risk populations.

“In this subdistrict, we had 26 *P. knowlesi* cases from 2022 to 2024. This year, only two cases have been reported so far.” — *Health Officer*

“During the dry season, macaques descend from the hills in search of food, which increases contact between humans and mosquitoes that can carry the *P. knowlesi* parasite.” — *Vector Control Staff*

“We typically find *P. knowlesi* in deep forest zones and the highland rubber plantations.” — *Village Health Volunteer*

“The number of *P. knowlesi* cases has dropped over the years, but we still see occasional infections among forest-goers and tourists, especially those who sleep without nets or walk through jungle trails.” — *Malaria Post Officer*

“Locally, we’ve observed seasonal spikes following increased macaque activity, usually toward the end of the rainy season.” — *Surveillance Officer*

#### 3.2.3. Control measures and public health responses to *P. knowlesi* malaria.

Although *P. knowlesi* is not explicitly targeted in Thailand’s national malaria elimination goals, it is treated as a notifiable human malaria infection and included in routine malaria surveillance and response activities. Participants described a comprehensive, multi-tiered response to *P. knowlesi* malaria control, aligned with national strategies and adapted to local epidemiological contexts. A central pillar of response was the 1-3-7 strategy; case notification within 1 day, investigation within 3 days, and targeted response within 7 days, often locally adapted to a 1-3-3 timeline for more rapid deployment. Other routine measures included indoor residual spraying (IRS), distribution of ITNs, active case detection, and vector surveillance in transmission hotspots. However, participants failed to acknowledge that these tools were originally designed to interrupt non-zoonotic transmission, and their effectiveness in controlling zoonotic malaria remains uncertain. IRS may have limited impact if infections occur outside households. Community health volunteers played an essential role in health education, symptom monitoring, and promoting personal protection, especially in remote or linguistically diverse populations. Health education efforts were typically intensified prior to the rainy season or periods of increased tourism.

“We conduct monthly screening in high-risk villages. Anyone with a fever is encouraged to get tested within three days.” — *Disease Surveillance Officer*

“We follow the 1-3-7 protocol strictly. After detecting a case, we screen a one-kilometer radius and spray indoors within a week.” — *Vector Team Leader*

“We educate villagers that *P. knowlesi* is different, it comes from monkeys via mosquitoes. That helps build community trust and cooperation.” — *Community Health Worker*

“During house visits, we not only distribute nets but also explain *P. knowlesi* transmission, stressing how forest visits and monkey contact raise the risk. Many people are surprised that malaria can come from animals.” — *Health Educator*

“In our area, health education is crucial. We train local volunteers and village leaders to share information about *P. knowlesi* malaria such as how it spreads, its symptoms, and ways to prevent it, at community meetings, especially ahead of the rainy season.” — *Health Promotion Officer*

#### 3.2.4. Operational challenges in controlling *P. knowlesi* malaria.

Despite strong control efforts, participants highlighted numerous operational barriers. One of the most frequently cited challenges was diagnostic uncertainty; *P. knowlesi* is morphologically similar to *P. malariae* under microscopy, making PCR confirmation necessary. However, PCR testing is expensive, logistically challenging in remote areas, and often results in several days’ delay. Some cases remain unconfirmed due to resource shortages or limited technical capacity.

Zoonotic transmission further complicates control. With macaques as natural reservoirs and no current system for wildlife surveillance or population control, participants expressed concern that conventional antimalarial strategies are insufficient. Since transmission is thought to primarily occur at night and deep in forested areas, household-based interventions such as IRS and bed nets were seen as only partially effective. Additional challenges identified included geographic isolation (e.g., islands, mountainous zones), language barriers among migrant communities, inconsistent public awareness, and declining support from external funding sources. These factors collectively hamper response speed, community engagement, and case follow-up.

“*P. knowlesi* often looks like *P. malariae* under the microscope. We often rely on PCR, but results take 3–5 days and the test is expensive.” — *Laboratory Officer*

“Some villagers still refuse to let us spray indoors or come for blood tests. They think malaria isn’t dangerous.” — *Health Promotion Officer*

“We can’t control monkey movements, but they’re central to *P. knowlesi* transmission. It’s a gap we’re not equipped to handle.” — *Regional Vector Specialist*

“Our biggest challenge is logistics. Many hotspots are in hard-to-reach areas, deep forest or offshore islands, which delays everything from diagnosis to spraying.” — *Malaria Control Staff*

“Sometimes, people self-medicate or hide their forest travel history, making it difficult to trace the source or interrupt transmission.” — *District Health Official*

## 4. Discussion

Raising awareness among community members is a critical component of effective malaria control, as it influences both adherence to prevention measures and treatment-seeking behavior. Prior studies have shown that higher levels of malaria awareness are associated with a reduced risk of infection and improved care-seeking, especially crucial for *P. knowlesi* malaria, which has a short erythrocytic parasite replication period of approximately 24 hours [29,33,34]. This study found encouraging levels of awareness among healthcare providers, with some understanding present among community members. However, awareness alone is not sufficient; tailored, multidisciplinary strategies are needed to challenge historical misconceptions and build trust in new tools and strategies. Existing responses to malaria in Thailand remain largely designed for non-zoonotic transmission and may have limited effectiveness for zoonotic malaria parasites such as *P. knowlesi*. This reflects a broader global issue, where elimination of *P. falciparum* and *P. vivax* has been achieved using standard tools, but *P. knowlesi* persists, as seen in Malaysia [14]. Importantly, while our findings suggest that providers are aware of *P. knowlesi* and familiar with relevant diagnostic methods, further evaluation is needed to assess their consistent availability and use in routine settings. Whether current surveillance systems are capturing the full extent of *P. knowlesi* transmission remains uncertain.

Healthcare providers consistently indicated that the recent increase in reported *P. knowlesi* cases in Thailand likely reflects improved diagnostic capacity, heightened awareness among frontline staff, and more systematic surveillance rather than a sudden epidemiological shift. This helps reconcile the apparent discrepancy between national data showing a rise in case numbers and local perceptions of declining caseloads. National increases between 2020 and 2023 were driven by short-term surges in a few provinces, whereas many areas, including the study sites, returned to low transmission by 2024 and 2025 [2]. These patterns suggest that *P. knowlesi* transmission in Thailand is highly focal and temporally variable, with improved detection contributing substantially to reported trends. Understanding this distinction is essential for interpreting surveillance data and tailoring appropriate response strategies.

In many rural settings across SEA, including in forest-adjacent communities in Thailand, males are often responsible for income generation, while females manage household and caregiving duties. Men may prioritize work outside the home and rely on their partners for health-related decisions. The majority of participants in this study were engaged in forest-related occupations, potentially placing them at heightened risk for *P. knowlesi* infection because of exposure in areas with active zoonotic transmission, such as forests, plantations, and other habitats where macaques and vectors coexist, and where there is limited access to conventional prevention methods like the use of insecticide-treated nets [19,35–37]. Healthcare providers echoed these concerns, citing the challenges of protecting forest-goers. Additionally, most malaria-related health education occurs during daytime working hours, and thus may not reach men who are away from home. These patterns likely contributed to the lower malaria awareness scores observed among males, a finding that contrasts with those reported in a previous study [27]. Male-targeted health promotion, flexible delivery (e.g., after-hours education), and indirect dissemination of information through mass media or intra-household communication may enhance reach and impact.

Transmission of *P. knowlesi* is concentrated in forested and remote settings, where ecological conditions foster high entomological indices and vectorial capacity [11]. In Thailand, malaria control and elimination activities, including Thailand’s 1-3-7 strategy, are carried out by local healthcare staff and VHVs [3]. In our study, many participants reported receiving health education from VHVs or healthcare workers. Although proximity to health facilities is often associated with better awareness, these facilities are typically situated in village centers rather than forested zones [38]. Given the sylvatic nature of *P. knowlesi* transmission, outreach activities often occur in transmission hotspots during case investigations, irrespective of residential proximity to health centers [39]. This may explain why participants who had received health education, regardless of their distance from health facilities, demonstrated higher awareness scores [28]. VHVs therefore remain a critical platform for extending malaria services into forested areas. Evidence from countries including Cambodia [40,41], Vietnam [42], and Myanmar [43] shows that community health workers can improve access to malaria diagnosis, treatment, and prevention among forest-goers by providing targeted counselling, distributing repellents or hammock nets, and supporting reactive surveillance. Adapting similar approaches for VHVs in Thailand could enhance coverage for high-risk, forest-exposed populations [15]. While Thailand’s malaria program is well established, these findings underscore the need to refine strategies to meet the operational and ecological challenges of zoonotic transmission.

A related challenge emphasized by healthcare providers is the difficulty of confirming *P. knowlesi* infections because the morphology of the parasite closely resembles that of *P. malariae*, making PCR confirmation necessary [8]. Strengthening diagnostic capacity will be essential for accurate surveillance. In the absence of nationwide PCR diagnostic coverage, practical options include expanding PCR capabilities at regional laboratories, such as ODPC and VBDC facilities, to reduce delays. Establishing simple triage criteria for sending samples for PCR, such as forest exposure or atypical microscopy findings, would improve efficiency. Additional training for microscopists to recognize features that warrant PCR referral and the use of emerging field-adapted molecular tools, such as LAMP assays, might further enhance species-specific diagnosis in remote settings [33]. Implementing these measures might help ensure timely confirmation of *P. knowlesi* cases and support targeted response activities in affected regions but will need to overcome difficult field conditions in order to be practically useful as point of care diagnostics. However, as the clinical management of *P. knowlesi* malaria is the same as for other types of malaria, subsequent confirmatory molecular testing can still play an essential role for surveillance.

Healthcare providers also noted that health education is commonly delivered during follow-up investigations, particularly after classifying reported *P. knowlesi* cases as indigenous or imported. As such, residents near case locations are more likely to receive targeted education. Although this reactive approach is beneficial, proactive strategies are needed to reach communities not immediately surrounding known case localities but who nonetheless live in high-risk environments. Currently, routine health education materials do not fully address zoonotic transmission involving non-human primates. Providers observed that many individuals were surprised to learn that malaria could be transmitted from monkeys. Future health education efforts should explicitly incorporate these findings, using evidence-based strategies tailored to identified risk factors, as supported by the PRECEDE–PROCEED model [44]. According to the WHO’s current malaria elimination definition [10], countries must demonstrate the interruption of indigenous transmission of the five primary human malaria parasite species, as well as negligible risk of infection by other species of *Plasmodiu*m, including zoonotic types such as *P. knowlesi*. Accordingly, stakeholders have increasingly advocated for the inclusion of *P. knowlesi* in national strategies, particularly given its wide distribution across SEA and potential to cause severe disease [14]. In Thailand, *P. knowlesi* remains a notifiable disease and is increasingly being recognized in policy discussions, even though it is not explicitly targeted within the national elimination framework.

Attitudes toward *P. knowlesi* malaria appear to be linked with overall awareness [18,29]. Individuals who perceive malaria as severe and potentially life-threatening may be more likely to engage with health information and discuss it within their social networks. Positive attitudes may also reflect prior exposure to health education, as described by healthcare providers. In our study, participants with higher attitude scores also had significantly greater awareness, in line with the Health Belief Model, which posits that perceived susceptibility and potential severity can drive preventive health behaviors [45]. Our sampling approach, which preferentially selected household heads or knowledgeable representatives, may also have influenced awareness and attitude scores. These individuals often hold decision-making roles, engage more frequently with community health workers, and may have greater exposure to malaria-related information than other household members. As a result, our estimates may modestly overrepresent community awareness. Future studies should consider including multiple members per household or exploring intra-household variations to better capture the full range of community understanding.

To our knowledge, this is the first study in Thailand to explore both community-level awareness and health provider perspectives on *P. knowlesi* malaria, addressing a critical gap in the existing literature. As the transmission pathway for *P. knowlesi* mirrors that of other mosquito-borne malaria parasite species, these findings may also inform broader malaria control strategies. However, the study has limitations. Data were collected only in the southern provinces of Thailand and may not be generalizable to other regions with differing ecological or sociocultural contexts. The cross-sectional design and reliance on structured questionnaires restricted the exploration of underlying reasons for low awareness among some groups. Although the study was conducted near international borders, only Thai citizens were included in the study. Future research should incorporate migrant and tourist populations, who may face additional barriers to malaria prevention and care [46]. Finally, while we included healthcare providers from the village to provincial levels, their perspectives may not fully capture challenges at higher administrative or policy determination and implementation levels.

## 5. Conclusion

This mixed-methods study provides novel insights into the awareness and attitudes toward *P. knowlesi* malaria among at-risk communities and healthcare providers in southern Thailand. Findings emphasize the need for tailored, context-specific health promotion strategies that target high-risk populations, especially forest-exposed males, and expand educational outreach beyond case-based responses. Integration of zoonotic malaria risks into routine health communication and alignment with multisectoral, one-health approaches will be essential for advancing malaria elimination goals in the region. As current control measures remain focused on non-zoonotic malaria, there is an urgent need to develop and adapt strategies that can effectively respond to the unique challenges posed by *P. knowlesi*. The findings resulting from this study reflect not only existing national priorities but also broader challenges for surveillance and control of zoonotic malaria across SEA.

## Supporting information

S1 Fig Overview of qualitative study participants. This figure presents the breakdown of participants included in the qualitative component of the study, organized by role and setting. It includes healthcare providers, malaria commanders, and other key informants engaged in malaria control and response efforts in the study area. The total number of participants is shown along with their classification.
(TIF)

S1 File Quantitative questionnaire. (DOCX)

S2 File Qualitative guidelines. (DOCX)

S1 Text Model specification for generalized linear regression. (DOCX)

S1 Data Dataset. (CSV)
