# Supplementary material for: Community awareness and health providers’ perspectives on zoonotic Plasmodium knowlesi malaria in Thailand: A mixed-methods assessment
Source: PLoS Negl Trop Dis. 2026 Feb 18;20(2):e0013891. doi: 10.1371/journal.pntd.0013891 (PMC12931881; doi:10.1371/journal.pntd.0013891)
Supplement: S1 File — (DOCX) [file pntd.0013891.s002.docx]

**S1 File. Quantitative questionnaire for community members**

1. **Socioeconomic information**

| 1 | How old are you? | __________________(years) |
| --- | --- | --- |
| 2 | Gender | - Male  - Female |
| 3 | Education level | - No formal/ primary education  - Primary school  - Secondary school  - College and above |
| 4 | Current occupation | - Farmers/agricultural workers  - Forest workers  - Gardeners  - Dependent  - Housewife  - Government employees  - Merchants  - Others (please specify)  ________________________ |
| 5 | Citizenship | - Thai  - Myanmar  - Malaysia  - Laos  - Cambodia  - Others (please specify)  _______________________ |
| 6 | Estimated annual income | _______________________(THB) |
| 7 | Number of family members | ______________________ |
| 8 | Relationship to household head | - Household head  - Husband/Wife  - Brother/Sister  - Son/Daughter  - Other relatives |
| 9 | Length of residence in current location | _______________________(years) |
| 10 | Distance to the nearest health facility | _______________________(miles) |
| 11 | Time to reach the nearest health facility | _______________________(minutes) |
| 12 | Have you had any previous experience with malaria? | - Yes  - No |
| 13 | If yes, how many years ago was your most recent malaria episode? | ______________________(years) |
| 14 | Have you ever participated in malaria-related health education? | - Yes  - No |
| 15 | Where do you get most of your health information about malaria?  (Select all that apply) | - Health professionals  - Community health workers  - Village health volunteers  - Radio/TV  - Internet/Social media  - Family/Friends  - Other (please specify)  ______________________________ |

**2. Awareness of *Plasmodium knowlesi* Malaria (Transmission, Risk and Prevention)**

1. Have you ever heard of *P. knowlesi* malaria, *Pk* malaria, Monkey malaria, or malaria transmitted from monkeys?
   - Yes
   - No

*(Note: If participants respond with a specific keyword (e.g., Plasmodium knowlesi, Pk malaria, Monkey malaria, or malaria transmitted from monkeys) at the beginning of the questionnaire, continue using that keyword consistently throughout the rest of the questions. Example: If the participant says they are familiar with the term "Monkey malaria," make sure you use "Monkey malaria" in all subsequent questions related to malaria.)*

1. Are you aware of any recent cases of *P. knowlesi* malaria in your community or nearby regions?
   - Yes
   - No
2. Have you ever seen long-tailed and pig-tailed macaques around your household or workplace? (Please refer to figures below)
   - Yes
   - No

***(Note:*** *We used reference images from Google and other relevant sources to help participants conveniently identify the mentioned types of monkeys. However, the images have been omitted here due to copyright restrictions.)*

1. Do you know of any specific groups or activities that are at higher risk of *P. knowlesi* malaria in your area? (Select all that apply)
   - Forest workers
   - Farmers
   - People living near forests
   - Children
   - Pregnant women
   - Older people
   - Others (please specify) ______________________________________
2. Do you consider yourself to be at risk of contracting *P. knowlesi* malaria?
   - Yes
   - No
3. Do you know how *P. knowlesi* malaria is transmitted? (Select all that apply)
   - Via mosquito bites
   - Direct contact with monkeys
   - Through contaminated water or food*
   - Living/working in the forest
   - Eating bananas/papayas*
   - Staying close to malaria patients
   - I don’t know*
   - Others (please specify) ______________________________________
4. Do you know any ways to protect yourself from *P. knowlesi* malaria? (Select all that apply)
   - Using mosquito nets
   - Wearing long-sleeved clothing
   - Applying mosquito repellents
   - Burning rubbish
   - Avoiding forested areas
   - Others (please specify) ______________________________________
5. On a scale of 1 to 5, how confident are you in your understanding of *P. knowlesi* malaria?
   - 1 (Not confident)
   - 2
   - 3
   - 4
   - 5 (Very confident)

**3. Perceptions about *P. knowlesi* Malaria**

[Please indicate your level of agreement with the following statements on a scale of 1 (Strongly Disagree) to 4 (Strongly Agree)]

| **#** | **Statements** | **Strongly disagree** | **Disagree** | **Agree** | **Strongly agree** |
| --- | --- | --- | --- | --- | --- |
|  |  | **1** | **2** | **3** | **4** |
| 1 | Knowlesi malaria is becoming a serious problem in my area. |  |  |  |  |
| 2 | Knowlesi malaria can have severe consequences for health. |  |  |  |  |
| 3 | Monkeys can transmit malaria through mosquito bites. |  |  |  |  |
| 4 | Knowlesi malaria cannot be prevented. * |  |  |  |  |
| 5 | Knowlesi malaria cannot be treated. * |  |  |  |  |
| 6 | Knowlesi malaria is not as dangerous as other types of malaria. |  |  |  |  |
| 7 | Health workers in my area are well-prepared to handle cases of knowlesi malaria. |  |  |  |  |
| 8 | The standard malaria prevention methods (e.g., mosquito repellents) are not effective against knowlesi malaria. * |  |  |  |  |
| 9 | Raising awareness about knowlesi malaria will help reduce its spread in the community. |  |  |  |  |

*Negative answers/ statements.
