# Supplementary material for: Community awareness and health providers’ perspectives on zoonotic Plasmodium knowlesi malaria in Thailand: A mixed-methods assessment
Source: PLoS Negl Trop Dis. 2026 Feb 18;20(2):e0013891. doi: 10.1371/journal.pntd.0013891 (PMC12931881; doi:10.1371/journal.pntd.0013891)
Supplement: S2 File — (DOCX) [file pntd.0013891.s003.docx]

**S2 File. Qualitative Guidelines for healthcare providers/ Village Health Volunteers**

1. **Demographics**

Age (years) ……………………………………..

Gender ……………………………………..

Years of service in current position ……………………………………..

Duty station ……………………………………..

**2. Qualitative questions**

2.1 Can you briefly describe the current trend of *Plasmodium knowlesi* malaria in your area, comparing the situation over the past three years?

……………………………………………………………………………………………………………………………………………………………………………………………………………………………………………………………………………………………………………………………………………………………………………………………………………………………………………………………………………………………………………………

2.2 Are there any specific measures or activities focused on controlling *P. knowlesi* malaria in your area? If so, what are they? Can you briefly explain these measures or activities?

……………………………………………………………………………………………………………………………………………………………………………………………………………………………………………………………………………………………………………………………………………………………………………………………………………………………………………………………………………………………………………………

2.3 What challenges or difficulties do you face when carrying out the activities mentioned above?

……………………………………………………………………………………………………………………………………………………………………………………………………………………………………………………………………………………………………………………………………………………………………………………………………………………………………………………………………………………………………………………
