# Supplementary material for: Community awareness and health providers’ perspectives on zoonotic Plasmodium knowlesi malaria in Thailand: A mixed-methods assessment
Source: PLoS Negl Trop Dis. 2026 Feb 18;20(2):e0013891. doi: 10.1371/journal.pntd.0013891 (PMC12931881; doi:10.1371/journal.pntd.0013891)
Supplement: S1 Text — (DOCX) [file pntd.0013891.s004.docx]

**Model specification for generalized linear regression**

**1. Model Formula (R Notation)**

The generalized linear model (GLM) used to identify factors associated with awareness scores was:

| awareness ~ age + sex + education + occupation + income_k +  family_members + relationship_to_householdhead +  length_of_residence + distance_facility + time_to_reach +  malaria_experience + malaria_he + attitudes +  factor(village)  # *Village was included as a fixed effect to account for site-level variation.* |
| --- |

**2. Full R Code Used for Model Estimation**

| **# GLM with robust SEs, adjusted for village as fixed effect**  glm_model <- glm(  awareness ~ age + sex + education + occupation + income_k +  family_members + relationship_to_householdhead +  length_of_residence + distance_facility + time_to_reach +  malaria_experience + malaria_he + attitudes +  factor(village),  data = data,  family = gaussian()  )  **# Robust standard errors**  coeftest(glm_model, vcov = vcovHC(glm_model, type = "HC1")) |
| --- |

**3. Interaction Models Tested**

| **Model 1: Distance to facility × Malaria health education**  glm_model1 <- glm(  awareness ~ age + sex + education + occupation + income_k +  family_members + relationship_to_householdhead +  length_of_residence + distance_facility + time_to_reach +  malaria_experience + malaria_he + attitudes +  factor(village) +  distance_facility * malaria_he,  data = data,  family = gaussian()  )  coeftest(glm_model1, vcov = vcovHC(glm_model1, type = "HC1"))  **Model 2: Education × Malaria health education**  glm_model2 <- glm(  awareness ~ age + sex + education + occupation + income_k +  family_members + relationship_to_householdhead +  length_of_residence + distance_facility + time_to_reach +  malaria_experience + malaria_he + attitudes +  factor(village) +  education * malaria_he,  data = data,  family = gaussian()  )  coeftest(glm_model2, vcov = vcovHC(glm_model2, type = "HC1"))  **Model 3: Sex × Occupation**  glm_model3 <- glm(  awareness ~ age + sex + education + occupation + income_k +  family_members + relationship_to_householdhead +  length_of_residence + distance_facility + time_to_reach +  malaria_experience + malaria_he + attitudes +  factor(village) +  sex * occupation,  data = data,  family = gaussian()  )  coeftest(glm_model3, vcov = vcovHC(glm_model3, type = "HC1")) |
| --- |

**4. Notes**

- Village was included as a fixed effect because only six purposively selected villages were studied; random-effects estimation is unreliable with very few clusters.
- Robust (HC1) standard errors were used to adjust for heteroskedasticity.
- All interaction models were non-significant and excluded from the final model.
